# A hub and spoke model to supply the Sicilian neurorehabilitation demand: effects on hospitalization rates and patient mobility

**DOI:** 10.3389/fpubh.2024.1349211

**Published:** 2024-03-20

**Authors:** Augusto Ielo, Angelo Quartarone, Rocco Salvatore Calabrò, Maria Cristina De Cola

**Affiliations:** IRCCS Centro Neurolesi Bonino Pulejo, Messina, Italy

**Keywords:** hub-and-spoke, patient mobility, neurorehabilitation, quality indicators, Pabón Lasso model

## Abstract

**Introduction:**

Cerebrovascular diseases in Sicily have led to high mortality and healthcare challenges, with a notable gap between healthcare demand and supply. The mobility of patients seeking care, both within and outside Sicily, has economic and organizational impacts on the healthcare system. The Hub and Spoke model implemented by the IRCCS Centro Neurolesi “Bonino-Pulejo” of Messina aims to distribute advanced neurorehabilitation services throughout Sicily, potentially reducing health mobility and improving service accessibility.

**Methods:**

The evaluation was based on calculating hospitalization rates, examining patient mobility across Sicilian provinces, and assessing the financial implications of neurorehabilitation admissions. Data from 2016 to 2018, covering the period before and after the implementation of the Hub and Spoke network, were analyzed to understand the changes brought about by this model.

**Results:**

The analysis revealed a significant increase in hospitalization rates for neurorehabilitation in the Sicilian provinces where spokes were established. This increase coincided with a marked decrease in interregional health mobility, indicating that patients were able to receive high-quality care closer to their residences. Furthermore, there was a decrease in both intra-regional and inter-regional escape rates in provinces within the Hub and Spoke network, demonstrating the network’s efficacy in improving accessibility and quality of healthcare services.

**Discussion:**

The implementation of the Hub and Spoke network substantially improved neurorehabilitation healthcare in Sicily, enhancing both accessibility and quality of care for patients. The network’s establishment led to a more efficient utilization of healthcare resources and balanced distribution of services. These advancements are vital steps toward equitable and effective healthcare delivery in Sicily.

## 1 Introduction

In 2019, the mortality rate due to cerebrovascular diseases of the brain in Sicily was 11.4 per 10,000 inhabitants, one the highest among Italy regions (1). This mortality estimate, together with the high rates of hospital discharge and drug consumption for these conditions, reflects the need to plan new strategies to improve the services provided by national (NHS) and regional health systems.

Patients with acquired brain injury in the post-acute phase may present with a vegetative state, a minimally conscious state, or severe disability. Neurorehabilitation improves the spontaneous recovery of these neurological disabilities in a short time through various approaches (2). However, Sicilian healthcare supply seems to be underestimated compared to demand; in fact, intensive rehabilitation is the field with the highest percentage of mobility to other regions (about 15%) (3).

Health mobility refers to the scenario in which patients move from their area of residence to seek medical care and treatment. The mobility can occur within the region (intraregional) or across the country (interregional). Intraregional mobility describes patients traveling within the boundaries of the region they reside in, while interregional mobility refers to traveling from one region to another, primarily seeking better treatment than the one provided in their home region. In Italy, about 7.6% of hospital admissions occurred during 2020 were interregional (4), monetized about 3.33 billion of euros as national health spending, with highly variable balances between northern and southern regions. Notably, Sicily was one of the Italian regions with the highest debit balance (€ 173.3 million) (5).

Political, economic, social and technological environments induce continuous changes in the healthcare scenario, by increasing its complexity. Thus, healthcare providers have to be able to navigate proficiently through different areas, including organization and supply of services (6).

Recently, hub-and-spoke organization design is taking place as alternative model for serving patients, centralizing the most advanced medical services at a single site (hub) and distributing basic services via secondary sites (spokes), routing patients needing more intensive services to the hub for treatment (7). Hence, the hub-and-spoke model yields a healthcare network consisting of a main site and one or more satellite sites. It offers opportunities to maximize efficiencies and effectiveness, besides being highly scalable, with satellites can be added as needed (8). For this purpose, a Hub and Spoke (HS) model was designed and tested from 2017 to 2022 by the IRCCS Centro Neurolesi “Bonino-Pulejo” of Messina, Sicily. This model, already described in previous studies (9, 10), has been created to export the skills and equipment on advanced robotic neurorehabilitation from Messina (Hub) to other cities of Sicily (i.e., Palermo, Catania, Trapani and Raguse) (Spokes), allowing to offer more suitable treatments to patients in their own cities, and reducing costs for inter- and intra-regional health mobility. Indeed, providing an “on-site” rehabilitation service is not only useful for the NHS, which would cut down on public expenditure, but also (and especially) for the users. Indeed, people may not have the chance to move outside their residence (often facing high costs for travel, overnight stays for any caregivers, etc.) or access to private rehabilitative facilities which are not always affiliated with the NHS. According to a recent report published by the National Institute of Statistics (ISTAT), in 2022, about 7% of the Italian population renounced necessary health care services considering them too expensive, or because of long waiting times for hospitalization (11).

In this study, we examined changes after the establishment of the HS network on demand and spending for hospitalization in neurorehabilitation in Sicilian provinces. Notably, we focused on variations from 2016 (i.e., before the introduction of the HS network) to 2018 (after the introduction of the HS network and before the COVID-19 pandemic). As secondary aim, the variations in hospital bed management for any Sicilian province were also investigated.

## 2 Methods

### 2.1 Outcome measures

To estimate the demand for hospitalization in neurorehabilitation we have considered hospitalization rates, hospital admission volumes, and patient migration across the Sicilian provinces. We also analyzed the performance of the HS network and estimated the healthcare spending (also called costs for brevity) due to neurorehabilitation admissions.

#### 2.1.1 Hospitalization rates in neurorehabilitation

Hospitalization rates in neurorehabilitation were calculated for each Sicilian province. The crude hospitalization rate was calculated by dividing the number of discharges in a certain geographic area by the total number of people residing in the same area (inhabitants). Standardized hospitalization rates were also calculated, in order to compare rates of Sicilian population over time. Thus, direct standardization (per 1,000 inhabitants) was employed to standardize rates using the 2006 Sicilian residents as the standard population (12). In addition, to remove the effect of the potential confounders, which might differ between populations and could distort the results, the hospitalization rates were standardized by age and sex using the following seven age classes: {18–24, 25–34, 35–44, 45–54, 55–64, 65–74, 75 and older}.

The formula used for calculating age- and sex-standardized hospitalization rates (SHR) is:


$$SHR=\frac{\sum_{i=1}^{7}\sum_{j=1}^{2}\left(n_{ij}\cdot r_{ij}\right)}{\sum_{i=1}^{7}\sum_{j=1}^{2}n_{ij}}$$

Where *n_ij_* is is the number of people in age class *i* and sex *j* within the standard population at-risk, and *r_ij_* is the age- and sex-specific crude rate of the population under consideration.

#### 2.1.2 Origin–destination flow data

Length of stay, i.e., the time elapsed between a patient’s hospital admittance and discharge were represented as proportion within origin–destination (OD) matrices, to allow the direct analysis of patients’ mobility across the Sicilian provinces. Similarly, hospitalization costs in euro (€) were represented within OD matrices.

#### 2.1.3 Escape and attraction indexes

Patient migration was measured by intra- and inter-regional escape and attraction indexes, in all four provinces of the H&S network:

- Intraregional escape (IRE) index was calculated dividing the number of discharges of patients residing in the province under review occurring in Sicily outside the same province, by the number of discharges of patients residing in the province under review.
- Interregional escape (ERE) index was calculated dividing the number of discharges of patients residing in the province under review occurring outside Sicily, by the total number of discharges of patients residing in the province under review.
- Intraregional attraction (IRA) index was calculated dividing the number of discharges of patients residing in Sicily outside the province under review, occurring within the same province, by the number of discharges occurred in the facilities placed within the province under review.
- Interregional attraction (ERA) index was calculated dividing the number of discharges of patients residing outside Sicily occurring in the province under review, by the discharges occurred in the facilities placed within the same province.

All the formulas used to calculate the escape and attraction indexes are reported in the Supplementary material.

#### 2.1.4 Hospitals performance

The Pabón Lasso model (13) was used to assess the relative performance of health facilities in terms of bed occupancy rate (BOR), bed turn over (BTO) and the average length of stay (ALOS):

- BOR indicator is calculated as the inpatient days of care for a given period divided by the number of beds available in that period.
- BTO is calculated as the number of discharges in a given time period divided by the total number of beds in the hospital during that time period.
- ALOS indicator is calculated as the sum of the number of days for all stays in a given time period divided by the number of discharges in that time period.

All the formulas used to calculate the indicators employed in the model are reported in the Supplementary material.

The model gathers together health facilities into 4 sectors representing different levels of productivity. In particular, on the x-axis is the BOR, while on the y-axis is the BTO. The diagonal lines represent the selected ranges of ALOS. Four sectors (1 to 4) were created using as intersection the midpoint calculated by aggregating the results of the 2 years under study (2016 and 2018). Thus, health facilities in the first sector (lower left) have low throughput (number of admission per bed) of patients and long periods where beds are empty; health facilities in sector two (upper left) treat a large number of patients per bed but have long periods when beds are unoccupied; health facilities in sector three (upper right) treat patients with high throughput and high occupancy; and health facilities in sector four have beds with low throughput and patients stay in health facilities longer. Therefore, the third sector represents the ideal situation, with a low proportion of unused beds.

### 2.2 Data source

We used the inpatients administrative database of the Regional Health System (RHS) from January 2016 to December 2018, which includes all hospital discharges in Sicily, as well as those of Sicilian residents in other Italian regions.

To identify hospitalizations for intensive neurorehabilitation, we used the version 24 of the diagnosis-related group codes (DRG) of International Classification of Diseases, Ninth Revision, Clinical Modification (ICD-9-CM) (14), and we selected only hospitalization with a DRG code belonging to the Major Diagnostic Category 1 (Diseases and Disorders of the Nervous System - MDC 1). We also filtered for clinical specialty and hospital disciplines codes, defined by the Italian Health Ministry (15). Notably, the selected codes were: 28 (spinal unit), 56 (functional recovery and rehabilitation) and 75 (neurorehabilitation; disorder of consciousness).

The following exclusion criteria were considered: patients aged under 18 years (as the HS network does not provide pediatric neurorehabilitation services); voluntarily discharged patients; short-term hospitalizations (0–1 day); incomplete records, i.e., records not including patient’s demographic characteristics, or spatial variables (region and province), or information concerning the hospitalization.

The number of available beds in each province in the years under study was also obtained from the administrative database of the RHS. To estimate the health spending for hospitalization in neurorehabilitation we used the inpatient hospital fee schedules of the Sicilian Region (16).

### 2.3 Statistical analysis

We compared the intra- and inter-regional escape and attraction indexes of the four Sicilian provinces within the HS network (Catania, Messina, Palermo and Trapani), before and after the HS network activation (2016 vs. 2018). Statistical analysis was performed by using the 4.2.3 version of the open-source software R. A *p* < 0.05 was considered as statistically significant. Results for categorical variables were expressed in frequencies and percentages. The Chi-test test for equality of proportions with continuity correction was used to assess for statistical differences in proportions.

## 3 Results

A total of 8,299 discharges records in rehabilitation units were included in this study (3,785 in 2016 and 4,514 in 2018). Of these, 7,087 (85.4%) were patients residing in Sicily admitted within their own region (3,182 in 2016 and 3,905 in 2018), 1,012 (12.2%) were Sicilian patients who opted for facilities outside Sicily (515 in 2016 and 497 in 2018), and 200 (2.4%) are patients residing outside Sicily that were admitted to Sicilian facilities (88 in 2016 and 112 in 2018). The mean age of the patients hospitalized in 2016 was 61.2 ± 17.5 years, whereas in 2018 was 62.1 ± 16.9 years.

### 3.1 Hospitalization rates in neurorehabilitation

**Figure 1:**
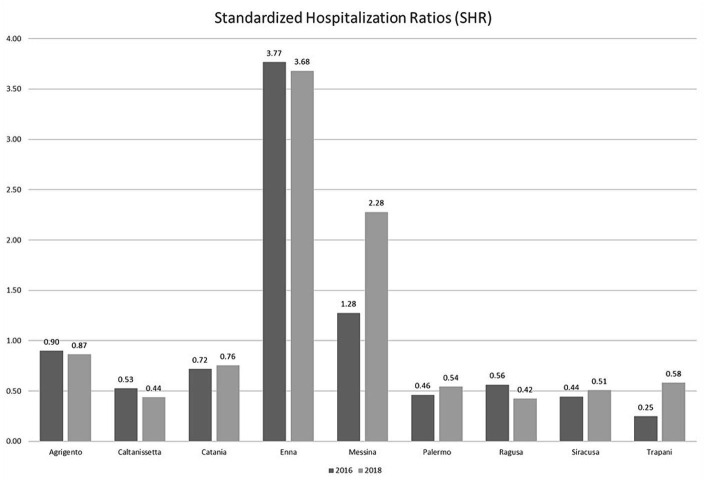
Standardized hospitalization rates (SHR). Neurorehabilitation hospitalization rates for each Sicilian province. Direct standardization (per 1,000 population) was employed to standardize rates by using the 2006 Sicilian population.

Figure 1 shows the standardized hospitalization rates in neurorehabilitation calculated for all Sicilian provinces, during the years 2016 and 2018. As shown in Table 1, an increase in the hospitalization rate is observed in the provinces where spokes were opened (Catania: from 0.72 to 0.76; Messina: from 1.28 to 2.28; Palermo: from 0.46 to 0.54; Trapani: from 0.25 to 0.58). The age- and sex-standardized hospitalization rates are reported in Supplementary material.

**Table 1:** 2016 / 2018 standardized hospitalization rates.

District	SHR	
	2016	2018
AG	0.90	0.87
CL	0.53	0.44
CT	0.72	0.76
EN	3.77	3.68
ME	1.28	2.28
PA	0.46	0.54
RG	0.56	0.42
SR	0.44	0.51
TP	0.25	0.58

SHR = standardized hospitalization rates; AG = Agrigento, CL = Caltanissetta, CT = Catania, EN = Enna, ME = Messina, PA = Palermo, RG = Ragusa, SR = Siracusa, TP = Trapani.

### 3.2 Origin–destination flow data

**Figure 2:**
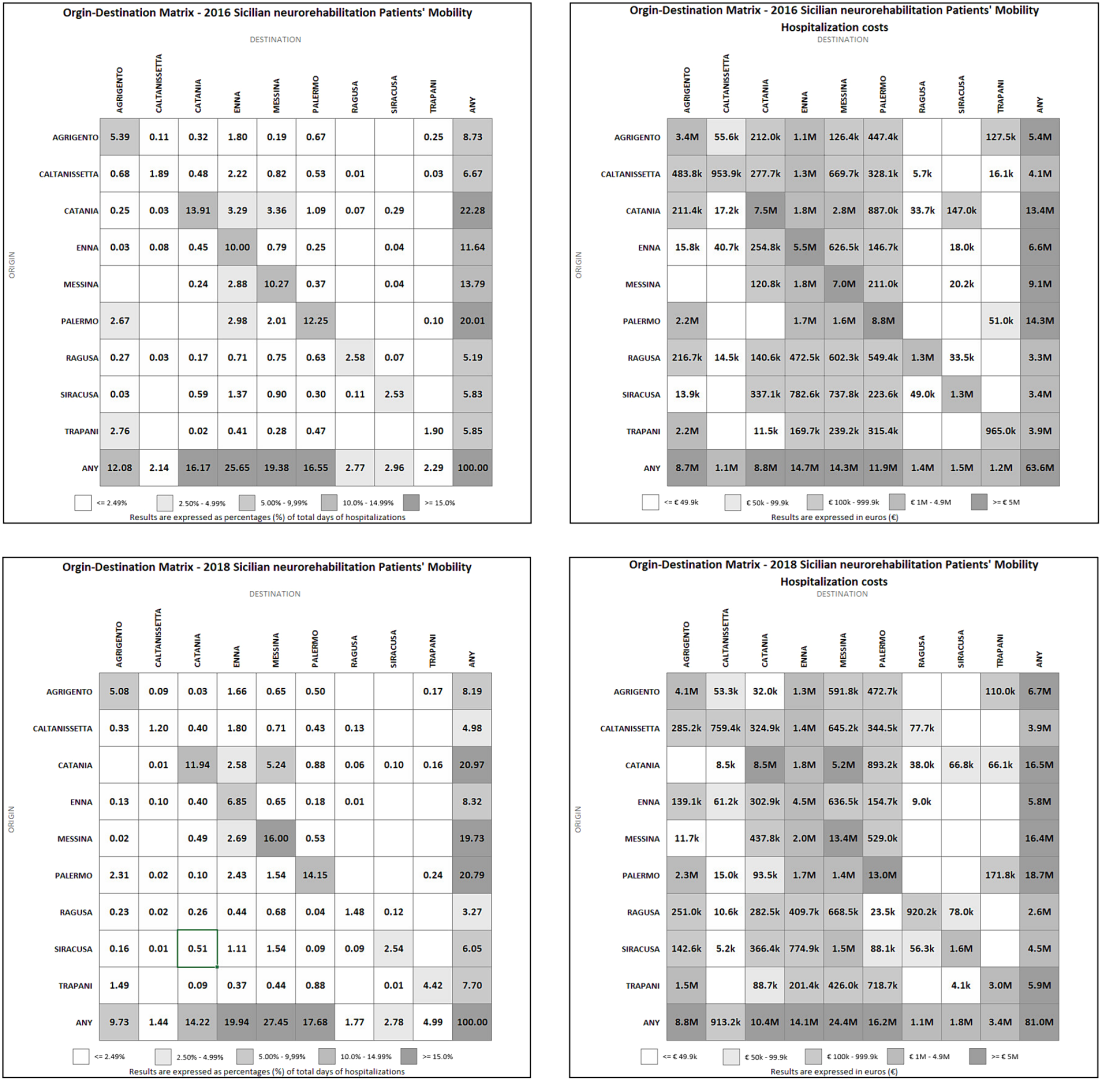
Origin–destination (OD) matrices of 2016 / 2018 neurorehabilitation patients’ mobility and hospitalization costs. k = thousands; M = millions.

The OD matrices of the length of stay and the total costs of hospitalizations are shown in Figure 2. An overall increase in the proportion of neurorehabilitation days of hospitalization between 2016 and 2018 was observed in the Sicilian provinces where the HS model was implemented (from 54.39 to 64.34%). Notably, we found an increase in patients’ mobility toward the provinces of Messina (from 19.38 to 27.45%), Palermo (from 16.55 to 17.68%) and Trapani (2.29 to 4.99%).

Regarding the costs of hospitalizations in neurorehabilitation, there was an increase in all provinces within the HS model: Catania from € 8,854,500 to € 10,428,700 (+ 17.78%); Messina from € 14,401,900 to € 24,468,000 (+ 69.89%); Palermo from € 11,908,600 to € 16,224,400 (+ 36.24%); Trapani from € 1,159,600 to € 3,347,900 (+ 188.71%). Furthermore, the costs increased from € 63,641,091 in 2016 to € 80,965,378 in 2018 (+ 27.2%) when we considered all hospitalizations for neurorehabilitation in Sicilian facilities. On the contrary, spending on out-of-region hospitalizations decreased about 16.5% from 2016 (€ 7,786,174.89) to 2018 (€ 6,500,172.00), and about 17.9% when the only provinces in HS model are considered.

### 3.3 Escape and attraction indexes

**Figure 3:**
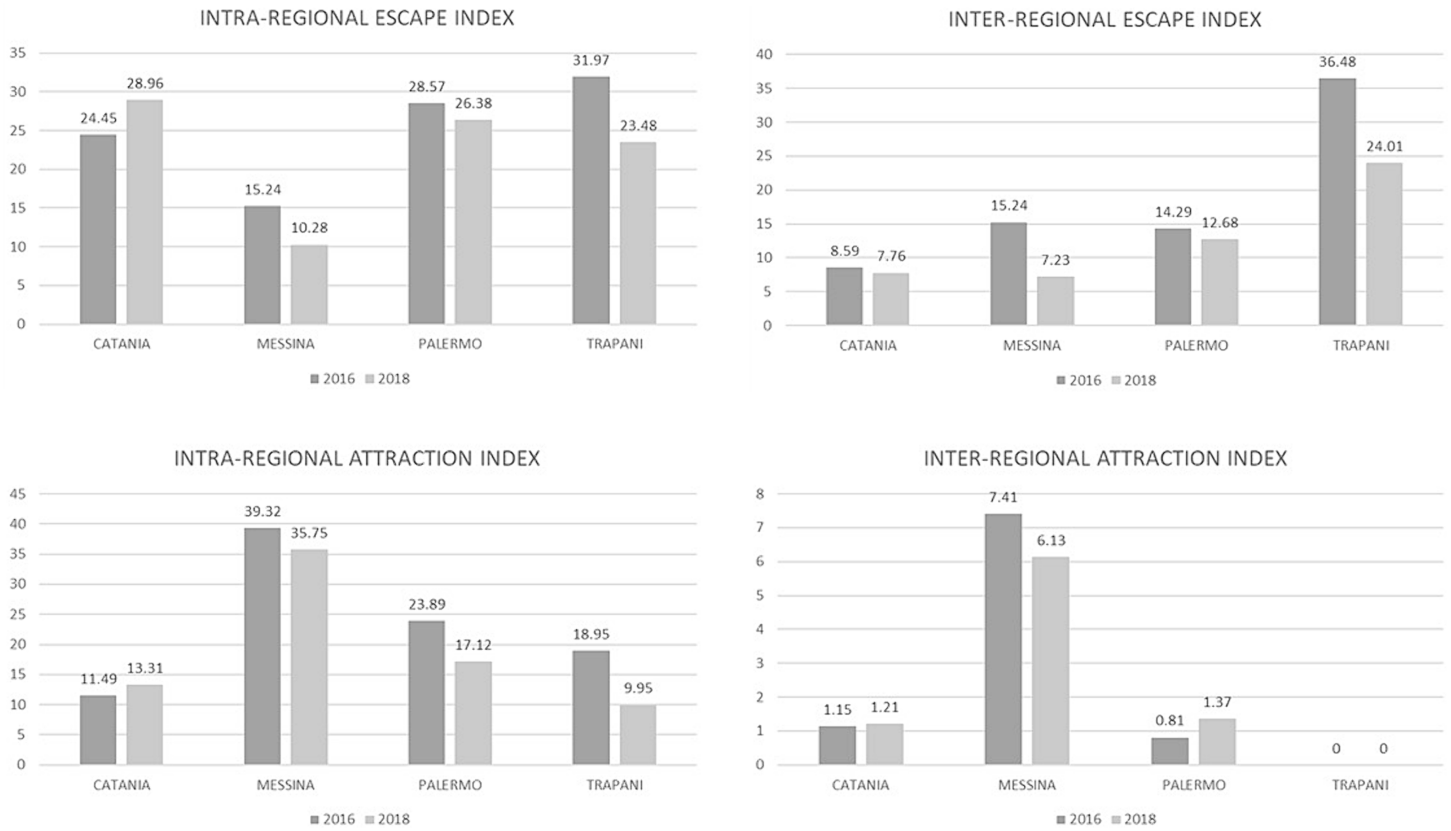
2016 / 2018 neurorehabilitation intra- and inter-regional escape and attraction indexes. Values are expressed in percentage (%).

The intra- and inter-regional escape and attraction indexes of the Sicilian provinces are included in Table 2. A graphical representation of the indexes for the provinces included in the HS model is showed in Figure 3. It is viewable that there was a statistically significant decrease from 2016 to 2018 of the escape index in the provinces of Messina (intra-regional from 15.24 to 10.28%, *p* = 0.007 and inter-regional from 15.24 to 7.23%, *p* < 0.001) and Trapani (intra-regional from 31.97 to 23.48%, *p* = 0.001 and inter-regional from 36.48 to 24.01%, *p* = 0.025), as well as in the province of Palermo, although did not reach the statistical significance (intra-regional from 28.57 to 26.38% and inter-regional from 14.29 to 12.68%).

**Table 2:** Statistical analysis of the intra- and inter-regional escape and attraction indexes between 2016 and 2018.

Escape index						
Province	IRE		*p*-value	ERE		*p*-value
	2016	2018		2016	2018	
Agrigento	30.00	28.13	0.490	15.29	12.26	0.888
Caltanissetta	47.23	54.69	0.104	12.18	11.72	0.978
Catania	24.45	28.96	0.031*	8.59	7.76	0.559
Enna	24.67	28.72	0.407	5.73	4.62	0.770
Messina	15.24	10.28	0.007**	15.24	7.23	< 0.001***
Palermo	28.57	26.38	0.385	14.29	12.68	0.417
Ragusa	26.56	32.63	0.204	15.77	16.32	0.983
Siracusa	33.57	37.50	0.349	17.33	15.06	0.509
Trapani	31.97	23.48	0.001**	36.48	24.01	0.025*

Attraction index						
Province	IRA		*p*-value	ERA		*p*-value
	2016	2018		2016	2018	
Agrigento	44.54	34.05	0.490	0.59	0.31	1
Caltanissetta	11.81	16.50	0.406	1.57	0	0.572
Catania	11.49	13.31	0.341	1.15	1.21	1
Enna	62.28	69.73	0.156	3.37	3.13	0.973
Messina	39.32	35.75	0.129	7.41	6.13	0.319
Palermo	23.89	17.12	0.007**	0.81	1.37	0.561
Ragusa	4.70	15.65	0.005**	2.01	0	0.345
Siracusa	17.07	10.58	0.106	0	1.06	0.542
Trapani	18.95	9.95	0.043*	0	0	NA

Values are expressed as percentages. Significant differences between 2016 and 2018 are highlighted with asterisks (*p < 0.05; **p < 0.01; ***p < 0.001). IRE = intra-regional escape index, ERE = inter-regional escape index, IRA = intra-regional attraction index, ERA = inter-regional attraction index.

### 3.4 Hospitals performance

**Figure 4:**
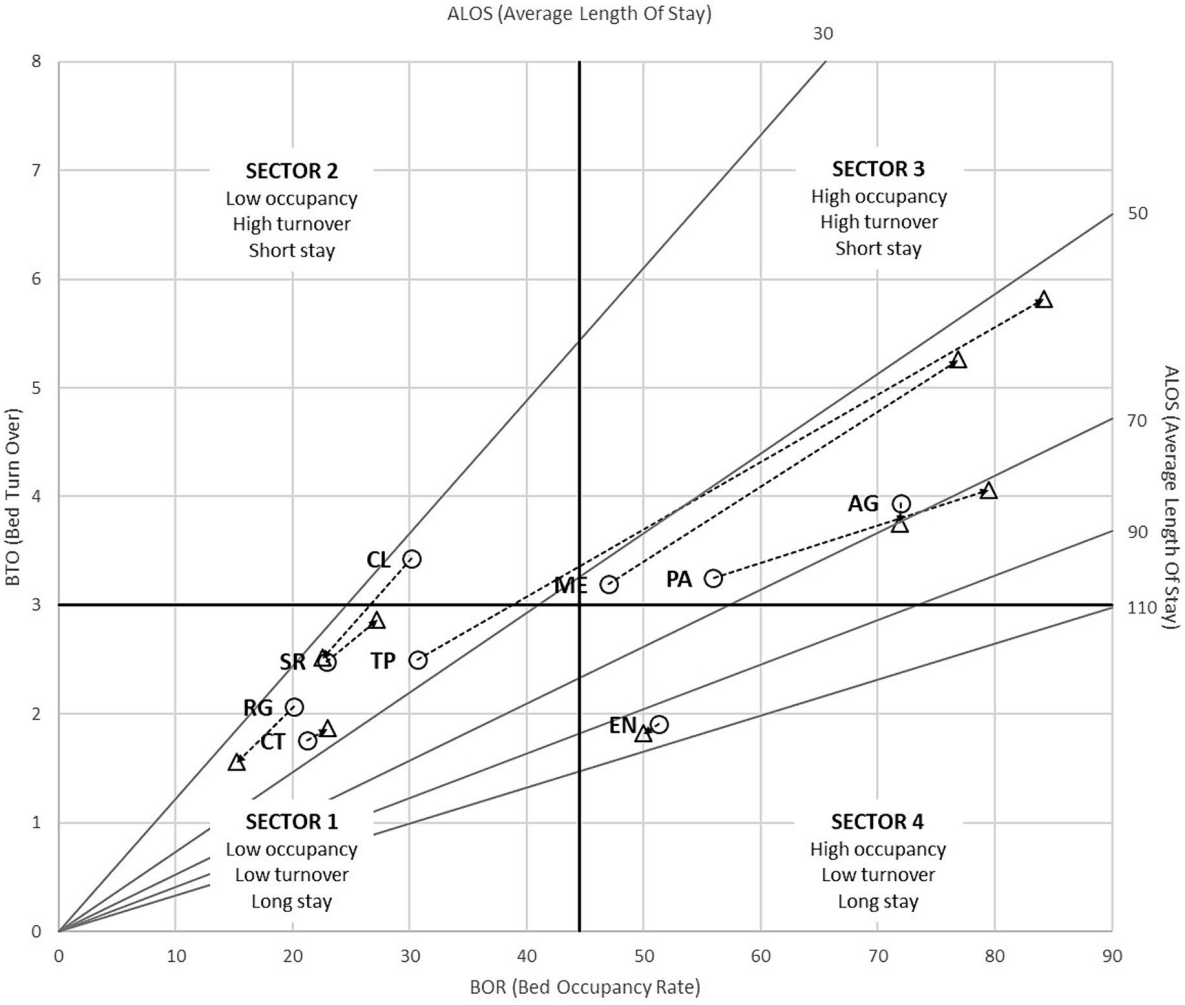
Pabón Lasso model of 2016 / 2018 neurorehabilitation care in Sicilian provinces. ◯ = 2016; △ = 2018; AG = Agrigento, CL = Caltanissetta, CT = Catania, EN = Enna, ME = Messina, PA = Palermo, RG = Ragusa, SR = Siracusa, TP = Trapani.

Figure 4 shows the hospital performances calculated using the Pabón Lasso model. Both years were illustrated in the same chart, connecting them with an arrow line to show the changes. The model shows improved performance in the provinces of Messina, Palermo and Trapani, all of which stand in the third sector in 2018. Notably, in 2016 the province of Trapani was located inside sector 1 (low occupancy, low turnover, long stay), while in 2018 has moved in sector 3 (high occupancy, high turnover, short stay).

## 4 Discussion

As far as we know, this is the first study, demonstrating how the use of a HS model may reduce intra and inter-regional mobility of neurorehabilitation inpatients in Sicily.

Health mobility offers patients the freedom to obtain health care from providers in different provinces, regions and even countries. It is essential to monitor patient choices to maintain quality standards and responsiveness in the health care system, which otherwise may suffer from geographic disparities in accessibility to quality and responsive health care (17).

In Italy, the Ministry of Health allocates funds to the regions in order to provide healthcare services and it monitors the activities of the regions. At local level, the delivery of health services is decentralized to a regional network of Local Healthcare Units (Aziende Sanitarie Locali, ASL), one per province, as well as independent public and private hospitals. In this organizational context, citizens who seek care at a public facility belong to the ASL of the province in which they live. However, they have the freedom to choose to receive health care from another ASL or another Italian region. This choice outlines the idea of active and passive interregional mobility, already recognized internationally (18, 19). In particular, active mobility indicates the attraction index of a region and identifies the healthcare services offered to non-resident citizens, while passive mobility identifies the healthcare services provided to citizens outside the region of residence (also known as escape index) (20).

Monitoring mobility flows is crucial in the legislative context to define the funds that will be allocated to a specific region or province for public health service (21). It is one of the main instruments to support business decisions in public administrations, and it helps in understanding and managing the delivery of healthcare services and in strategizing for long-term sustainability of health systems, as well as enables effective decision-making on health management policies (22). In the last decades, cross-border patient mobility has been a subject of interest by policy-makers and academics, not only internationally (23, 24) but also within national borders (25), and hospitalization rates have gained wide acceptance as indicators for policy choices. Variations in hospitalization rates for selected conditions are being used as indicators of the effectiveness of care in small areas (26). In addition, hospitalization rates are associated with multiple factors describing the individual and his/her ecology, including personal income and relative community wealth, race, residence, age, and sex, as well as the relationship between these factors and the incidence of the underlying disease. This makes it possible in many contexts to study the association between the characteristics of a local system of care and rates of preventable hospitalization. Thus, low hospitalization rates indicate better health care efficiency, both as the effectiveness of community services and as a reduction in the inappropriate use of hospitalization. However, an increase in hospitalization rates may reflect improved access to health care, especially if that increase coincides with the growth of treatment sites where patients can receive the services they need to meet their needs (27). Our findings show an increase in the hospitalization rate for neurorehabilitation in the provinces where spokes were opened, as well as an overall increase in neurorehabilitation admissions in almost all provinces, especially in the provinces of Messina and Trapani. The increase in hospitalization rates might reflect an enhancement in the utilization of healthcare services, suggesting that HS network is successfully addressing previously unmet medical needs within the community. In fact, patients had better access to specialized neurorehabilitation services that were previously less accessible or unavailable in their province. The increased hospitalization rate has led to a significant reduction in passive mobility, with a decrease in intraregional and interregional escape rates in the provinces within the HS network. This may confirm the improvement in the accessibility of healthcare services offered by the facilities belonging to the HS network. In addition, our findings might be insightful for other regions and healthcare contexts. As we know, the success of the HS network in enhancing accessibility and reducing patient mobility mirrors the objectives of established continuity of care networks in Northern Italy (10).

Changes in patient mobility before and after the implementation of the HS network resulted in a consequent increase in public spending. After all, the economic impact due to the implementation of a new integrated healthcare systems may vary depending on the specific context, as observed in other studies (28). Interestingly, the increase in spending is within the region itself, while spending on out-of-region mobility decreased by 16 percent, and as much as 18 percent in the provinces of the HS model. This might correspond to better management of health care funds by the RHS, and cost and burden savings for patients. They no longer have to travel long distances to receive specialized care, which can be more convenient and less stressful for them and their families (29, 30). Indeed, the successful implementation of the HS model presents a framework for the integration of neuro-rehabilitative Diagnostic, Therapeutic and Care Pathways (DTCP), aiming to further enhance the efficiency and effectiveness of patient care within this area. In addition, the HS network facilitates patient management, improving the regional neurorehabilitation health service in terms of integration and accessibility (10).Performance and efficiency enhancement are among the crucial concerns of policy-makers and managers of health care, nowadays. Factors such as effectiveness and efficiency of each RHS, presence of Reference Centers for specific diseases, waiting lists, diagnostic services and availability of treatments, and perceived or real quality of assistance, might influence patients’ mobility (20). For this reason, we also considered the performance of facilities belonging to the HS network. Pàbon Lasso findings show that hospitals in the HS network have improved their efficiency in bed management, a crucial aspect in the context of increasing demand for health care. This may also reflect an improvement in the quality of care, as well as shorter length of stays and higher turnover may indicate more effective treatments and faster recovery times for patients.

Two are the main limitations of this study: (i) the lack of a complete economic evaluation of the HS network, considering all costs to implement the services offered by the network. Unfortunately, we do not have economic information needed to carry it out. However, the effectiveness results found prompted the incorporation of the HS model into the Sicilian territorial network. To date, Trapani and Palermo spokes are part of the Sicilian RHS. (ii) The lack of a longer follow-up. Observation of mobility over a longer period may or may not confirm the results obtained. However, the HS network project has recently been closed, and the COVID-19 pandemic has definitely affected hospitalization volumes and rates between 2019 and 2022, especially because these are planned hospitalization and not in emergency. In our opinion, it is necessary to wait for a wash-out period to eliminate any influence due to the pandemic. Other limitations are the potential for selection bias in the data, the reliance on retrospective data analysis, and the lack of information on other variables that could influence the outcomes.

## 5 Conclusion

This study provides a comprehensive analysis of the impact of the Hub and Spoke (HS) network implementation on neurorehabilitation healthcare in Sicily. The HS network’s implementation led to increased hospitalization rates, particularly in provinces with new spokes, indicating improved access to specialized neurorehabilitation services. This increase has correspondingly reduced the need for inter-regional health mobility, allowing patients to receive quality care closer to their home. Additionally, the decrease in both intra-regional and inter-regional escape rates highlights the network’s effectiveness in enhancing healthcare service accessibility and quality. The improved hospital performance, as shown by the Pabón Lasso model, underscores the operational efficiency achieved in patient care and bed management. Overall, the HS network has successfully addressed unmet medical needs within the community, contributing to more efficient healthcare resource utilization and promoting better health outcomes in Sicily. Future studies could focus on examining patient mobility patterns to uncover spatial–temporal dynamics and service-specific aspects of healthcare usage as well as to explore the factors driving patient movement for healthcare services.

## Data availability statement

The data analyzed in this study were obtained from the Department of Health of the Sicilian Region, under the restrictions of non-public dissemination. Requests for access to these datasets should be addressed to Maria Cristina De Cola, mariacristina.decola@irccsme.it.

## Author contributions

AI: Conceptualization, Formal analysis, Investigation, Methodology, Writing – original draft. AQ: Funding acquisition, Supervision, Writing – review & editing. RC: Methodology, Supervision, Writing – review & editing. MC: Conceptualization, Data curation, Methodology, Writing – original draft.
