# Supplementary material for: A hub and spoke model to supply the Sicilian neurorehabilitation demand: effects on hospitalization rates and patient mobility
Source: Front Public Health. 2024 Mar 20;12:1349211. doi: 10.3389/fpubh.2024.1349211 (PMC10987749; doi:10.3389/fpubh.2024.1349211)
Supplement: Supplementary file 1 [file Table_1.docx]

A Hub and Spoke model to supply the Sicilian neurorehabilitation demand: effects on hospitalization rates and patient mobility

Augusto Ielo^1^, Angelo Quartarone^1^, Rocco Salvatore Calabrò^1*^, Maria Cristina De Cola^1^

^1^IRCCS Centro Neurolesi “Bonino Pulejo”, Messina, Italy

*** Correspondence:**Rocco Salvatore Calabrò
roccos.calabro@irccsme.it

# Table 1: Intra- and extra-regional escape and attraction indexes formulas

| **Indicator** | **Formula** |
| --- | --- |
| IRE | $IRE=\frac{D_{POP}}{D_{P}}\times100$ |
| ERE | $ERE=\frac{D_{POR}}{D_{P}}\times100$ |
| IRA | $IRA=\frac{D_{ROP}}{D_{F}}\times100$ |
| ERA | $ERA=\frac{D_{ROR}}{D_{F}}\times100$ |

LEGEND:

IRE = intra-regional escape index

ERE = extra-regional escape index

IRA = intra-regional attraction index

ERA = extra-regional attraction index

D_POP_ = number of discharges of patients residing in the province under review occurring in Sicily outside the same province

D_POR_ = number of discharges of patients residing in the province under review occurring outside Sicily

D_P_ = number of discharges of patients residing in the province under review

D_ROP_ = number of discharges of patients residing in Sicily outside the province under review

D_ROR_ = number of discharges of patients residing outside Sicily occurring in the province under review

D_F_ = number of discharges occurred in the facilities placed within the province under review
