# Supplementary material for: A hub and spoke model to supply the Sicilian neurorehabilitation demand: effects on hospitalization rates and patient mobility
Source: Front Public Health. 2024 Mar 20;12:1349211. doi: 10.3389/fpubh.2024.1349211 (PMC10987749; doi:10.3389/fpubh.2024.1349211)
Supplement: Supplementary file 2 [file Table_2.docx]

A Hub and Spoke model to supply the Sicilian neurorehabilitation demand: effects on hospitalization rates and patient mobility

Augusto Ielo^1^, Angelo Quartarone^1^, Rocco Salvatore Calabrò^1*^, Maria Cristina De Cola^1^

^1^IRCCS Centro Neurolesi “Bonino Pulejo”, Messina, Italy

*** Correspondence:**Rocco Salvatore Calabrò
roccos.calabro@irccsme.it

# Table 2: Hospital performance indicators formulas

| **Indicator** | **Formula** |
| --- | --- |
| BOR | $BOR=\frac{d_{S}}{N_{B}\times N_{d}}\times100$ |
| BTO | $BTO=\frac{D}{N_{B}}$ |
| ALOS | $ALOS=\frac{d_{S}}{D}$ |

LEGEND:

BOR = bed occupancy rate

BTO = bed turn over

ALOS = average length of stay

D = number of discharges

d_S_ = days of stay

N_B_ = total number of beds

N_d_ = total number of days
