# Supplementary material for: A hub and spoke model to supply the Sicilian neurorehabilitation demand: effects on hospitalization rates and patient mobility
Source: Front Public Health. 2024 Mar 20;12:1349211. doi: 10.3389/fpubh.2024.1349211 (PMC10987749; doi:10.3389/fpubh.2024.1349211)
Supplement: Supplementary file 3 [file Table_3.docx]

**A Hub and Spoke model to supply the Sicilian neurorehabilitation demand: effects on hospitalization rates and patient mobility**

**Augusto Ielo^1^, Angelo Quartarone^1^, Rocco Salvatore Calabrò^1*^, Maria Cristina De Cola^1^**

^1^IRCCS Centro Neurolesi “Bonino Pulejo”, Messina, Italy

*** Correspondence:**Rocco Salvatore Calabrò
roccos.calabro@irccsme.it

**Table 3: 2016 / 2018 neurorehabilitation age- and sex-standardized hospitalization ratios**

| **PROVINCE** | **SEX** | **SHR** | | | | | | | |
| --- | --- | --- | --- | --- | --- | --- | --- | --- | --- |
|  |  | **AGE CLASS** | | | | | | | **TOTAL** |
|  |  | **18-24** | **25-34** | **35-44** | **45-54** | **55-64** | **65-74** | **75-99** |  |
| **2016** | | | | | | | | | |
| AG | M | 0.033 | 0.099 | 0.033 | 0.162 | 0.286 | 0.318 | 0.175 | 1.106 |
|  | F | 0.018 | 0.013 | 0.069 | 0.108 | 0.154 | 0.198 | 0.149 | 0.710 |
| CL | M | 0.000 | 0.012 | 0.011 | 0.034 | 0.106 | 0.198 | 0.142 | 0.504 |
|  | F | 0.000 | 0.000 | 0.000 | 0.031 | 0.056 | 0.155 | 0.307 | 0.549 |
| CT | M | 0.022 | 0.028 | 0.032 | 0.084 | 0.122 | 0.239 | 0.231 | 0.758 |
|  | F | 0.003 | 0.016 | 0.020 | 0.038 | 0.074 | 0.185 | 0.348 | 0.684 |
| EN | M | 0.971 | 0.441 | 0.440 | 0.107 | 0.269 | 0.294 | 0.124 | 2.647 |
|  | F | 0.218 | 0.829 | 1.255 | 1.231 | 0.551 | 0.361 | 0.329 | 4.774 |
| ME | M | 0.047 | 0.108 | 0.176 | 0.202 | 0.291 | 0.352 | 0.260 | 1.437 |
|  | F | 0.030 | 0.027 | 0.080 | 0.132 | 0.259 | 0.291 | 0.312 | 1.131 |
| PA | M | 0.024 | 0.032 | 0.056 | 0.082 | 0.137 | 0.159 | 0.074 | 0.564 |
|  | F | 0.013 | 0.030 | 0.021 | 0.043 | 0.092 | 0.093 | 0.078 | 0.372 |
| RG | M | 0.000 | 0.021 | 0.037 | 0.030 | 0.095 | 0.176 | 0.278 | 0.638 |
|  | F | 0.018 | 0.010 | 0.000 | 0.021 | 0.116 | 0.136 | 0.194 | 0.495 |
| SR | M | 0.008 | 0.008 | 0.014 | 0.045 | 0.131 | 0.090 | 0.248 | 0.543 |
|  | F | 0.000 | 0.000 | 0.007 | 0.016 | 0.064 | 0.121 | 0.145 | 0.352 |
| TP | M | 0.000 | 0.000 | 0.013 | 0.032 | 0.052 | 0.152 | 0.069 | 0.318 |
|  | F | 0.000 | 0.000 | 0.000 | 0.014 | 0.027 | 0.053 | 0.090 | 0.184 |
| **2018** | | | | | | | | | |
| AG | M | 0.007 | 0.044 | 0.042 | 0.056 | 0.226 | 0.332 | 0.193 | 0.900 |
|  | F | 0.013 | 0.062 | 0.054 | 0.099 | 0.198 | 0.271 | 0.137 | 0.833 |
| CL | M | 0.000 | 0.024 | 0.059 | 0.034 | 0.034 | 0.155 | 0.144 | 0.451 |
|  | F | 0.000 | 0.000 | 0.023 | 0.008 | 0.046 | 0.143 | 0.209 | 0.429 |
| CT | M | 0.009 | 0.032 | 0.058 | 0.066 | 0.149 | 0.240 | 0.262 | 0.815 |
|  | F | 0.005 | 0.016 | 0.021 | 0.031 | 0.085 | 0.182 | 0.364 | 0.703 |
| EN | M | 0.832 | 0.585 | 0.507 | 0.096 | 0.213 | 0.325 | 0.179 | 2.738 |
|  | F | 0.401 | 0.583 | 1.117 | 1.276 | 0.509 | 0.390 | 0.256 | 4.531 |
| ME | M | 0.108 | 0.156 | 0.311 | 0.358 | 0.535 | 0.726 | 0.467 | 2.660 |
|  | F | 0.033 | 0.047 | 0.172 | 0.242 | 0.369 | 0.463 | 0.610 | 1.934 |
| PA | M | 0.023 | 0.036 | 0.075 | 0.089 | 0.173 | 0.182 | 0.090 | 0.667 |
|  | F | 0.011 | 0.021 | 0.070 | 0.066 | 0.078 | 0.096 | 0.089 | 0.430 |
| RG | M | 0.000 | 0.000 | 0.000 | 0.053 | 0.091 | 0.181 | 0.196 | 0.522 |
|  | F | 0.000 | 0.010 | 0.009 | 0.050 | 0.021 | 0.086 | 0.159 | 0.336 |
| SR | M | 0.000 | 0.008 | 0.007 | 0.017 | 0.115 | 0.271 | 0.181 | 0.599 |
|  | F | 0.000 | 0.000 | 0.007 | 0.059 | 0.090 | 0.114 | 0.152 | 0.423 |
| TP | M | 0.000 | 0.016 | 0.021 | 0.079 | 0.179 | 0.266 | 0.107 | 0.668 |
|  | F | 0.012 | 0.037 | 0.007 | 0.048 | 0.098 | 0.142 | 0.164 | 0.508 |

LEGEND:

SHR = standardized hospitalization ratios

AG = Agrigento

CL = Caltanissetta

CT = Catania

EN = Enna

ME = Messina

PA = Palermo

RG = Ragusa

SR = Siracusa

TP = Trapani
